# Optimization of photon beam energy in aperture‐based inverse planning

**DOI:** 10.1120/jacmp.v10i4.3012

**Published:** 2009-09-03

**Authors:** Jason St‐Hilaire, Caroline Sévigny, Frédéric Beaulieu, Luc Gingras, Daniel Tremblay, Luc Beaulieu

**Affiliations:** ^1^ Département de radio‐oncologie Centre Hospitalier Universitaire de Québec Québec Canada; ^2^ Centre de recherche en cancérologie de l'Université Laval Centre Hospitalier Universitaire de Québec Québec Canada

**Keywords:** aperture‐based IMRT, energy optimization, intensity modulation, inverse planning, optimization

## Abstract

Optimal choice of beam energy in radiation therapy is easy in many well‐documented cases, but less obvious in some others. Low‐energy beams may provide better conformity around the target than their high‐energy counterparts due to reduced lateral scatter, but they also contribute to overdosage of peripheral normal tissue. Beam energy was added as an optimization parameter in an automatic aperture‐based inverse planning system. We have investigated a total of six cases for two sites (prostate and lung), representative of deep‐seated and moderately deep‐seated tumors. For one case for each site, different numbers of beam incidences were considered. The other cases for each site were optimized using a fixed number of incidences. Four types of plans were optimized: 6 MV, 23 MV, and mixed energy plans with one or two energies per incidence. Each plan was scored with a dose‐volume cost function. Cost function values, number of segments, monitor units, dose‐volume parameters, and isodose distributions were compared. For the prostate and lung cases, energy mixing improved plans in terms of cost function values, with a more important reduction for a small number of beam incidences. Use of high energy allowed better peripheral tissue sparing, while keeping similar target coverage and sensitive structures avoidance. Low energy contribution to monitor units usually increased with the number of beam incidences. Thus, for deep‐seated and moderately deep‐seated tumors, energy optimization can produce interesting plans with less peripheral dose and monitor units than for low energy alone.

PACS numbers: 87.55.de, 87.55.dk, 87.56.N‐

## I. INTRODUCTION

Intensity‐modulated radiation therapy (IMRT) is an excellent technique for the planning of treatments requiring the delivery of complex dose distributions. However, this method is very demanding in both human and material resources. In order to reduce the burden due to planning, quality assurance, and delivery, and to obtain similar results, some authors have proposed inverse planning algorithms that are able to reproduce one or many of the different aspects of IMRT while improving what is currently done in three‐dimensional conformal radiotherapy (3D‐CRT).^(^ ^1^ ^–^ ^6^ ^)^ In this study, we use an aperture‐based IMRT (AB‐IMRT) system, called Ballista.^(^ ^7^ ^,^ ^8^ ^)^ This system can simultaneously optimize beam orientations (couch and gantry angles), wedge filters, collimator angles, and field weights, and includes multiobjective optimization^(9)^ while using multileaf collimator (MLC) apertures based on an anatomic segmentation. It is directly coupled to the Pinnacle^3^ treatment planning system (Philips Medical Systems, Andover, MA), via the PinnComm interface, for anatomy import, dose calculation (heterogeneous superposition/convolution algorithm), and plan review. Ballista has proved useful for many complex cases such as gynecologic malignancies^(^ ^10^ ^,^ ^11^ ^)^ or prostate cancer treatments involving hip prostheses.^(8)^ Its potential for head and neck cancers has also been investigated.^(12)^

Design of a treatment plan often brings up the question of beam energy selection. While in well‐documented cases the optimal choice is easy, in some others it is less obvious, especially for deep‐seated tumors. Low‐energy beams provide better conformity around the target than their high‐energy counterparts due to reduced lateral electronic scatter, but they also induce higher entry dose. The idea of energy optimization was discussed by Söderström et al.^(13)^ His study showed that, for deep‐seated targets, higher energies are profitable when using “classical” uniform dose delivery but lose relevance when using modulation, such as in beamlet‐based IMRT. Also, the use of a large number of beam portals diminishes the need to optimize the energy. A study by Pirzkall et al.^(14)^ has also revealed that with photon‐based IMRT, the use of an energy of 6 MV with less than nine fields may result in a dose increase in regions distant to the target volume, even though the usual dosimetric indicators suggest a good conformity of high dose to the target itself and a good sensitive structure sparing. With a greater number of fields, the difference between dose distributions at 6, 10, and 18 MV disappears.

A few investigations have been conducted about the relevance of the optimal energy choice for specific anatomic sites. Head and neck cancers are an example where low energies are preferable. These cancers can be treated indifferently with an energy between ^60^Co and 6 M V, because these energies all produce similar dose distributions^(^ ^13^ ^,^ ^15^ ^)^ and no difference was found in outcome or toxicity.^(16)^ Prostate cancers, being deep‐seated, are more likely to require high photon energy for treatment, because of its greater tissue penetration.^(17)^ However, intensity‐modulated plans at 6 MV can be equivalent to 18 MV for prostate treatments in large patients, provided that proper inverse planning techniques are adopted.^(18)^ Also, according to Fox et al.,^(19)^ 18 MV plans actually require more fluence to provide similar quality target coverage. Finally, most of the debate around the choice of beam energy involves lung cancer treatments. Because the lungs are a low‐density medium, dose calculation accuracy is more difficult to achieve due to greater lateral dose disequilibrium. Bloomquist et al.^(20)^ have evaluated, with a Monte Carlo method, the advantages of using very high beam energy (50 MV) over low energy (6 MV). They concluded that optimal solutions would require the use of both high‐ and low‐energy beams. Wang et al.,^(21)^ Weiss et al.,^(22)^ and Madani et al.^(23)^ independently mentioned that high energy should not be excluded a priori, but that its use must be carefully weighted to overcome lateral disequilibrium problems and potential dose calculation inaccuracies. This suggests that an adequate use of high energy for lung cancer necessitates optimization through inverse planning, as well as a dose calculation algorithm that correctly models radiation transport in heterogeneous media.

At our institution, beamlet‐based IMRT treatments are conducted at 6 M V. However, for AB‐IMRT treatment plans such as those produced by Ballista, intensity modulation and segments are limited; a gain from energy optimization could, therefore, be obtained.

The purpose of this paper is to introduce two methods for the inclusion of energy in the optimization process: the optimization of a *virtual angle*, and the duplication of fields with a different energy for each copy. The performance of these approaches is shown for six cases covering two anatomical sites: prostate and lung. To our knowledge, no similar multi‐energy inverse planning optimization studies have been conducted in the past. Various numbers of incidences (one case per site) and energy patterns (all cases) were analyzed. Advantages, disadvantages, and relevance of each method are discussed.

## II. MATERIALS AND METHODS

Details about Ballista and its many components are described elsewhere.^(9)^ However, it is worth mentioning that the implementation of energy optimization takes advantage of two different algorithms: (1) a simulated annealing (SA) engine, and (2) a bound‐constrained quasi‐Newton (BCQN) engine. The former is used in the optimization of beam configurations (table, gantry, collimator, and wedge angles), while the latter finds optimal beam weights.

### A. Anatomy‐based apertures

**Figure 1:**
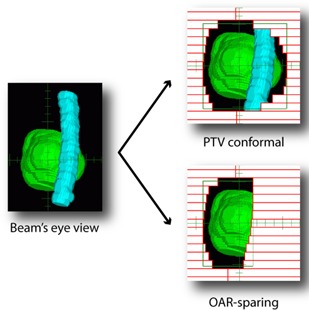
Generation of conformal and derived segments from a beam's eye view projection of segmented structures. (PTV: green, OAR: blue)

For each beam incidence, the AB‐IMRT system automatically generates a series of segments. The first one is conformal to the planning target volume. Then, starting from this conformal field, additional subfields are created in which a user‐specified sensitive structure is blocked by the MLC leaves. Each field is attributed an independent weight, thus allowing all of them to contribute to the total dose distribution according to the beam geometry. An example of this aperture generation process is shown in Fig. 1.

### B. Single‐energy optimization

The SA engine is used to explore the solution space by varying stochastically the couch (α), gantry (β), collimator (γ), and wedge (θ) angles. SA mimics the cooling of a crystalline system and the rearrangement of its atoms in a lowest energy level configuration. By analogy, this method allows the minimization of a cost function **ϕ(*x*)** by optimizing a set *x* of independent variables. The temperature T of the system is to be reinterpreted as a dimensionless control parameter, decreasing with each iteration. SA has first been described by Metropolis et al.^(24)^ and applied to optimization processes by Kirkpatrick et al.^(25)^ Ballista uses a modified version of this algorithm, called fast simulated annealing (FSA), where the temperature and the maximum change in variables decrease more rapidly, as described by Szu and Hartley.^(26)^ Implementation of the Metropolis criterion for configuration acceptance and the Lorentzian generating function for step size choice follows what has been previously described by Rosen et al.^(27)^

The discrete concept of energy levels does not apply to the aforementioned formalism the same way it applies to the angles α, β, Γ, and θ. Thus, an *energy angle* (ε) was introduced as an additional, independent degree of freedom to the system. This virtual angle is allowed to vary between 0° and 360°. In order to translate this value to an actual energy level, the expanded solution space is partitioned in a number of subspaces equal to the number of energy levels $N_{E}$. When the angle falls within a given partition, it is associated to the corresponding energy. This type of optimization implies that all segments of a given gantry angle have to be at a single energy: it is thus called *single‐energy optimization* (SEO). Because it is implemented at the FSA step, it only influences the number of configurations and has little impact over computational burden – the main effect being that more iterations may be needed to obtain the optimal solution.

### C. Multiple‐energy optimization

In Ballista, each incidence is subdivided in a series of segments according to an anatomy‐based segmentation. Each segment *j* of a beam *i* contributes to the dose distribution proportionally to its weight $w_{i,j}$. All segment weights are part of the optimal solution ***w*** where, for $N_{B}$ beams and $N_{S}^{i}$ segments per beam:

(1) $$\begin{array}{ll} w=\left\{w_{i,j}\right\} & i=1,2,\;\ldots ,\;N_{B}\\ & j=1,2,\;\ldots ,\;N_{S}^{i} \end{array}$$

The beam weight optimization implemented in Ballista solves the minimization problem for ***w*** using a BCQN algorithm (OPT++ 2.0, Sandia National Laboratories, Livermore, CA).

The second type of energy optimization actually begins before the BCQN engine is called. In order to further improve the treatment plans, more than one energy is made available to each segment. This approach, called *multiple‐energy optimization* (MEO), resides in the duplication of each segment according to the number of energy levels $N_{E}$. Therefore, for the *i*th segment of the *i*th beam, the segment $S_{i,j}$ becomes a set of $N_{E}$ segments $\left\{S_{i,j,k}\right\}$, where $k=1,\ldots N_{E}$. To each duplicated segment $S_{i,j,k}$ is associated a weight $w_{i,j,k}$, that is optimized by the BCQN engine. Each segment can therefore include contributions from more than one energy of photons and counterbalance their strengths and weaknesses. This approach is however more demanding in terms of CPU and memory, because the number of variables is increased $N_{E}$‐fold.

### D. Coverage margins

A margin is added to the anatomic apertures depending on the width of the beam penumbra, but also on the contribution of other beams. Low‐energy beams show smaller penumbras than high‐energy beams, which means that the coverage margin has to be greater for the second case.

Since for a given incidence, more than one energy (ε) can be used by Ballista, coverage margins for each of the $N_{E}$ energies available can be different. Therefore, for each set of {α, β, γ, θ} values, $N_{E}$ patterns of leaf positions are created, each corresponding to an energy level. In this study, coverage margins of 0.4 and 0.6 cm were assigned to 6 MV and 23 MV beams, respectively. Shielding margins can also be applied when covering an OAR for a subfield; however, while the option is available, no such margin was used here.

### E. Dose‐volume cost function and optimization parameters

The optimization process for angles {α, β, γ, θ, ε} is driven by a cost function (CF), a numerical method for quantifying plan quality. Its value has to be minimized to find an optimal plan. In the present case, a dose‐volume CF was used, based on the work by Wu and Mohan.^(28)^ Instead of only considering the dose received by a given structure, the partial volume irradiated at this dose is taken into account. The evaluation of dose‐volume criteria can be expressed by the following cost function:

(2) $$\Phi =\sum_{j=1}^{\Omega }\frac{1}{N_{j}C_{j}}\sum_{i=1}^{N_{j}}\sum_{c=1}^{C_{j}}dv_{\text{jic}}$$

where ${\text{dv}}_{\text{jic}}$ is the score of the *c*th dose‐volume optimization parameter (OP) for structure *j* evaluated at dose point $i,\;C_{j}$ and $N_{j}$ are respectively the number of dose‐volume OPs and dose points for structure *j*, and Ω is the number of structures (targets and OARs) considered. The term ${\text{dv}}_{\text{jic}}$ equals:

(3) $$dv_{\text{jic}}=\eta_{jc}\cdot H(D_{1}-D_{ji})\cdot H(D_{ji}-D_{2})\cdot {\left(D_{1}-D_{ji}\right)}^{2}$$

for a coverage OP $\left(V(>D_{1})>V_{1}\right)$, and

(4) $$dv_{\text{jic}}=\eta_{jc}\cdot H(D_{ji}-D_{1})\cdot H(D_{2}-D_{ji})\cdot {\left(D_{ji}-D_{1}\right)}^{2}$$

for a hot spot $OP(V(>D_{1})<V_{1})$, where $D_{1}$ is the dose OP for the partial volume $V_{1},\;D_{2}$ is the actual dose received by $V_{1},\;\eta_{\text{jc}}$ is the importance factor associated with the *c*th OP of structure *I*, and *H(x)* is the step function defined as

(5) $$H(x)=\{\begin{array}{ll} 1, & \text{if}\;x>0\\ 0, & \text{if}\;x\leq 0 \end{array}$$

**Figure 2:**
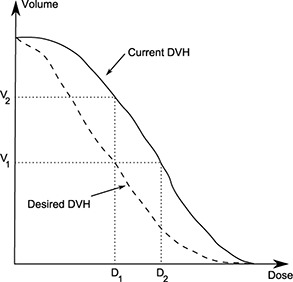
Schematic representation of the parameters used in the dose‐volume cost function. (Figure inspired by Wu and Mohan.^(28)^

Thus, the penalty score for each point *i* is proportional to the square of the difference between the dose $D_{\text{ji}}$ and the OP, when $D_{\text{ji}}$ has a value between $D_{1}$ and $D_{2}$. A schematic representation of the determination of parameters $V_{1},V_{2},D_{1},$, and $D_{2}$ is shown in Fig. 2.

Dose‐volume criteria have the advantage of providing more flexibility to the optimization process and greater control over dose distributions than dose criteria. In fact, dose OPs can be considered a subset of dose‐volume OPs in which the volume is set to an extreme value (0 or 100%, depending on the situation).

### F. Dose calculation

Dose calculation is performed using the collapsed‐cone superposition/convolution algorithm in Pinnacle^3^, correcting for heterogeneities. This is a well‐established dose calculation method that shows excellent agreement with the TG‐23 test package,^(29)^ with 96% of comparison points being within ±2% of tabulated values.^(30)^ During optimization, for each beam orientation chosen by the FSA engine at any given iteration, the Ballista system calculates (fast convolution, dose grid resolution of 6 mm) and stores the dose distribution or loads the corresponding dose file. After optimization, the treatment is reconstructed in Pinnacle^3^ with the adaptive convolution method, with a dose grid resolution of 4 mm.

### G. Plan evaluation

Comparison of all the plans generated by the AB‐IMRT system was done using their respective isodose distributions and dose‐volume histograms (DVHs). Typical dosimetric parameters used were the maximum and mean doses ($D_{\max}$ and $D_{\text{mean}}$), the volume receiving at least *x* Gy $\left(V_{\text{xGy}}\right)$ and the dose received by *y*% of a volume $\left(D_{y\%}\right)$. Conformity indices (CIs) and homogeneity indices (His) were calculated using the following expressions, where $V_{p}$ is the volume of the *p*% isodose and $V_{\text{PTV}}$ is the volume of the PTV:

(6) $$\text{CI}(p)=\frac{V_{p}}{V_{\text{PTV}}}$$

(7) $$\text{HI}=\frac{D_{5\%}}{D_{95\%}}$$

The definition of CI(*p*) implies that both the PTV and the volume covered by the *p*% isodose have to be overlapping, as stated by the International Commission on Radiation Units and Measurements report 62.^(31)^ Plans were also evaluated by comparing CF values, number of segments, and number of monitor units (MUs) for each energy used, either 6 MV or 23 MV.

### H. Cases studied

Two anatomic sites were investigated to test the implementation of energy optimization: prostate and lung – representative of deep‐seated and moderately deep‐seated target volumes. All plans mentioned below were generated as if to be delivered with a 29 leaf‐pair MLC mounted on a Primus linear accelerator (Siemens Medical Solutions, Erlangen, Germany), able to produce 6 MV and 23 MV bremmstrahlung beams.

#### H.1 Prostate cancer

Three cases of prostate cancer were studied. These are later called cases PI, P2 and P3. The clinical target volume (CTV) was contoured by a radiation oncologist on computed tomography (CT) images. The planning target volume (PTV) was defined as a 0.5 cm expansion of the CTV and was prescribed to receive 76 Gy (38 fractions of 2 Gy). The rectum, the bladder, and the femoral heads were contoured by an experienced dosimetrist.

In addition to the anatomic contours, two conformity zones were created. A transition zone, defined as an annular region surrounding the PTV from 0.5 cm to 3.5 cm in the lateral and anteroposterior directions and from 0.8 cm to 3.8 cm in the cranio‐caudal direction, was used to limit maximum dose in order to obtain tighter isodose conformity. A rest‐of‐body region was also defined, extending from the transition zone's outer contour to the external contour of the patient. This region was mainly used to monitor dose near the surface, in order to avoid peripheral overdosages. Other groups have used this strategy, notably Wu and Mohan.^(28)^ The values we used were determined by experience.

Dose‐volume OPs are shown in Table 1. The goal was to encompass the entire PTV with the 100% isodose (76 Gy), while having the 105% isodose (79.8 Gy) cover less than 5% of that volume. A series of dose‐volume OPs were defined for the rectum, with doses ranging from 50 to 75 Gy. These values were inspired from a literature review on the correlation of dose‐volume parameters and rectum complications.^(^ ^32^ ^–^ ^39^ ^)^ In the present study, values used in the optimization were actually lower than those from the review. The femoral heads’ maximum dose OP values were taken from Emami et al.^(40)^ They were considered separately, meaning that each femoral head contributed to the CF value in Eq. 2. In addition to a maximum dose constraint on the bladder, a low‐dose constraint was added in order to limit the volume of high‐dose regions. These could not be completely avoided since the bladder overlaps partially with the PTV. The maximum doses for the transition and rest‐of‐body regions were set to 68.4 Gy (90% of the prescription dose) and 50 Gy, respectively.

The plans generated by Ballista consisted in a given number of coplanar beams (gantry angle optimized), with an anatomic segmentation defined by the PTV and the rectum. Four plans were optimized: two monoenergetic plans at 6 and 23 MV, a SEO plan, and a MEO plan. For case P1, configurations with three, four, five, and seven beams were explored, for a total of 6, 8, 10, and 14 segments available (twice as much for MEO). For cases P2 and P3, only the five beam configurations were optimized. The initial positions of the gantry were those of an equidistant configuration. The initial temperature for the FSA engine was set to 120, the initial width of the Lorentzian distribution to 180, and the slowdown parameter to 2. Optimization stopped upon reaching 1500 iterations or when the CF value remained within 5% for 50 iterations.

**Table 1:** Dose‐volume optimization parameters and importance factors in the cases of prostate cancer.

*Target or Organ*	*Parameter*		*Value*	*Importance Factor*
Planning Target Volume	$V_{76\text{Gy}}$	>	100%	100
	$V_{79.8\text{Gy}}$	<	5%	100
Bladder	$D_{\max}$	<	70 Gy	15
	$V_{20\text{Gy}}$	<	50%	15
Rectum	$V_{50\text{Gy}}$	<	60%	25
	$V_{55\text{Gy}}$	<	58%	25
	$V_{60\text{Gy}}$	<	45%	25
	$V_{65\text{Gy}}$	<	40%	25
	$V_{70\text{Gy}}$	<	20%	25
	$V_{75\text{Gy}}$	<	5%	25
Left Femoral Head	$D_{\max}$	<	52 Gy	10
Right Femoral Head	$D_{\max}$	<	52 Gy	10
Transition Zone	$D_{\max}$	<	68.4 Gy	10
Rest‐of‐Body Region	$D_{\max}$	<	50 Gy	10

Note: Rectum optimization parameters are more restrictive than constraints found in the literature review.

#### H.2 Lung cancer

The other three cases studied were non‐small cell lung cancers (L1, L2 and L3). The GTV and CTV were first contoured on the set of CT images by a radiation oncologist, with the help of CT images taken with a contrast agent. A PTV was then defined as a 1.0 cm expansion of the CTV. The OARs contoured were the healthy lungs (total lung volume minus the PTV),^(41)^ the spinal cord, the heart, and the esophagus. A 0.5 cm expansion was added to the spinal cord to form its planning at risk volume (PRV). Transition and rest‐of‐body regions were also created, with the same definition as for the prostate case.

Table 2 presents the dose‐volume OPs used for this particular site. The prescription to the PTV was 60 Gy, in 30 fractions of 2 Gy. The 95% isodose (57 Gy) was planned to encompass the entire PTV. We decided to specify an OP for the volume of heart receiving 15 Gy ${(V}_{15\text{Gy}})$ in order to limit mean dose. A maximum dose of 48 Gy was set to the spinal cord PRV. The maximum dose for the transition and the rest‐of‐body regions were set to 90% (54 Gy) and 80% (48 Gy) of the prescription dose, respectively. The healthy lungs were considered as a single volume.

The plans produced by Ballista consisted in coplanar beam configurations of segments conforming to the PTV and shielding the contralateral lung and the spinal cord PRV. Four plans were optimized: two monoenergetic plans at 6 and 23 MV, a SEO plan and a MEO plan. For cases L1, configurations with three, four, five, and seven beams were studied with the same four energy optimization patterns, corresponding to 9, 12, 15 and 21 segments available (twice as much for MEO). For cases L2 and L3, four beam incidences were used. The initial configurations were equidistant. The initial temperature was set to 120, the initial width to 180, and the slowdown parameter to 1. The maximum number of iterations was set to 1500, or up to the point where the CF value remained within 5% for 50 iterations.

**Table 2:** Dose‐volume optimization parameters and importance factors in the cases of lung cancer.

*Target or Organ*	*Parameter*		*Value*	*Importance Factor*
Planning Target Volume	$V_{57\text{Gy}}$	>	100%	20
	$V_{60\text{Gy}}$	>	90%	20
Esophagus	$V_{55\text{Gy}}$	<	28%	10
Heart	$V_{15\text{Gy}}$	<	15%	8
Healthy Lungs	$V_{10\text{Gy}}$	<	30%	16
	$V_{20\text{Gy}}$	<	22%	16
Spinal Cord PRV	$D_{\max}$	<	48 Gy	50
Transition Zone	$D_{\max}$	<	54 Gy	5
Rest‐of‐Body Region	$D_{\max}$	<	48 Gy	5

### I. convergence speed

By adding an energy angle to the simulated annealing process in SEO or doubling the number of variables in MEO, one should expect an impact on the convergence speed of both algorithms. The maximum number of iterations and the convergence criterion of the FSA engine were decided upon analysis of the evolution of the cost function value along the optimization. For this study, the five coplanar beam configurations of the prostate case were analyzed.

For the BCQN engine, the algorithm was allowed about 600 iterations per descent in order to find the minimum of the cost function. When convergence was not obtained for a given descent, a new random set of initial weights was chosen and the descent was redone. For each iteration of the FSA engine, a minimum of two successful descents of the BCQN engine was required to confirm convergence.

### J. nomenclature

Beam configurations will be mentioned in the text by using abbreviations of the type “*NC – E*“, where *N* is the number of beam incidences, *C* stands for coplanar, and *E* is the energy optimization pattern (either 6, 23, SEO, or MEO), when applicable. For example, “5C‐SEO” means a configuration with five coplanar incidences, with one energy per incidence. In some figures, for clarity purposes, the acronyms SEO and MEO are further abbreviated to S and M.

## III. RESULTS

### A. convergence of the FSA engine

**Figure 3:**
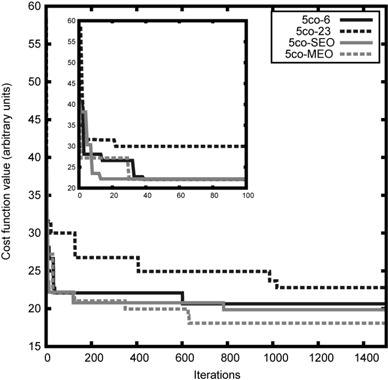
Lowest cost function value obtained after each iteration, for different optimizations of 5‐coplanar beam configuration for the prostate case P1. The smaller figure is a zoom on the first 100 iterations.

Figure 3 shows the evolution of the lowest CF value after each of the 1500 iterations of four 5C optimizations for the prostate case. It can be seen that within the last 500 iterations, the lowest CF value did not change much, justifying our choice for the maximum number of iterations. These optimizations took 93.7 min (6 MV), 87.4 min (23 MV), 138.5 min (SEO), and 410.5 min (MEO) on a SunBlade 2000 workstation (two 900 MHz CPUs; Sun Microsystems, Santa Clara, CA). Each iteration involves a superposition/convolution dose calculation, which is slower but more accurate than other algorithms such as a pencil‐beam method. The number of angles is doubled (ten instead of five) for the SEO plan. The number of beam weights is also doubled (20 instead of 10) for the MEO plan.

### B. Prostate case

For case P1, coplanar configurations with 3, 4, 5, or 7 beam portals were investigated. The CF values, number of segments and number of MUs for the final, optimized plans are presented in Table 3. For each configuration, energy optimization resulted in a lower CF value, except for the 7C‐SEO plan. When comparing the best monoenergetic and the best polyenergetic plans, this improvement was of 10.3% (3C), 12.0% (4C), 11.7% (5C), and 3.1% (7C). When considering only monoenergetic plans, plans at high energy were better, in terms of CF value, than their low‐energy counterparts for 3C, while the converse was true for 4C, 5C, and 7C. Low‐energy plans always used more MUs than high‐energy plans. Also, in energy optimization, 6 MV contributions to MUs tended to increase when adding a beam incidence, while the total number of MUs was between those of the two monoenergetic cases.

**Table 3:** Cost function (CF) values, number of segments (Seg), and number of monitor units (MU) for the energy optimization in the prostate case P1.

			*6 MV*		*23 MV*		*Total*	
*Confg.*	*Trial*	*CF*	*Seg*	*MU*	*Seg*	*MU*	*Seg*	*MU*
3C	6 MV	49.1705	4	414 (100.0%)			4	414
	23 MV	33.1939			5	326 (100.0%)	5	326
	SEO	29.7820	2	102 (30.9%)	2	228 (69.1%)	4	330
	MEO	29.8994	2	107 (30.4%)	4	245 (69.6%)	6	352
4C	6 MV	23.0966	7	437 (100.0%)			7	437
	23 MV	26.2134			7	329 (100.0%)	7	329
	SEO	21.0778	2	57 (17.0%)	5	278 (83.0%)	7	335
	MEO	20.3175	5	221 (58.0%)	3	160 (42.0%)	8	381
5C	6 MV	20.6272	6	433 (100.0%)			6	433
	23 MV	22.7879			7	332 (100.0%)	7	332
	SEO	19.8573	7	294 (75.2%)	1	97 (24.8%)	8	391
	MEO	18.2240	5	250 (65.8%)	4	130 (34.2%)	9	380
7C	6 MV	18.3730	11	439 (100.0%)			11	439
	23 MV	22.4733			8	333 (100.0%)	8	333
	SEO	18.5486	10	337 (80.4%)	1	82 (19.6%)	11	419
	MEO	17.7971	8	306 (75.6%)	4	99 (24.4%)	12	405

**Figure 4:**
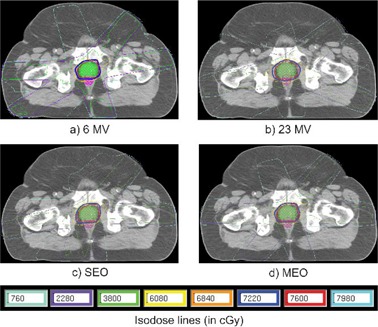
Isodose distributions for a 5‐coplanar beam configuration and for each type of energy optimization, in the prostate case P1. Structures shown are the PTV (green) and the rectum (pink).

**Figure 5:**
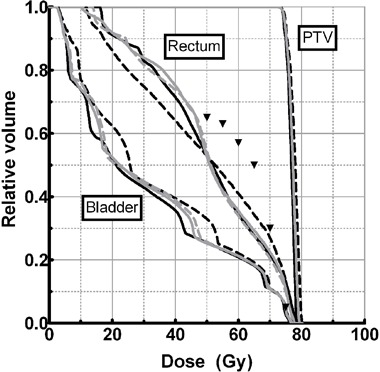
Dose‐volume histograms for a 5‐coplanar beam configuration and for each type of energy optimization, in the prostate case P1. Black triangles mark the dosimetric constraints based on the literature review. (solid black: 6 MV; dashed black: 23 MV; solid grey: SEO; dashed grey: MEO)

Isodose inspection shows that reduced peripheral dose was obtained when optimizing the energy and when increasing the number of beams. Figure 4 presents isodose distributions for 5C configurations, allowing comparison of peripheral dose. They are quite similar on the transversal plane shown, the $V_{95\%}$ and $V_{100\%}$ isodoses covering slightly more volume for the 23 MV plan, although the difference does not seem to be clinically important. The DVHs presented in Fig. 5 show the similarity between doses deposited in the PTV for each plan. Rectum DVHs vary more, but they respect all but one of the dosimetric thresholds based on the previously‐mentioned literature review.

For 3C configurations (not shown), 6 MV beams produced regions near the surface of more than 105% of the prescription dose. Such regions were not present when optimizing energy, even if low‐energy beams contributed to one third (30.9% SEO, 30.4% MEO) of total monitor units. Maximum dose to the rest‐of‐body region went from 78.1 Gy with 6 MV beams to 65.3 and 65.2 Gy for SEO and MEO. The advantage was less important when increasing the number of beam portals: maximum peripheral doses became progressively similar between the four plans.

**Figure 6:**
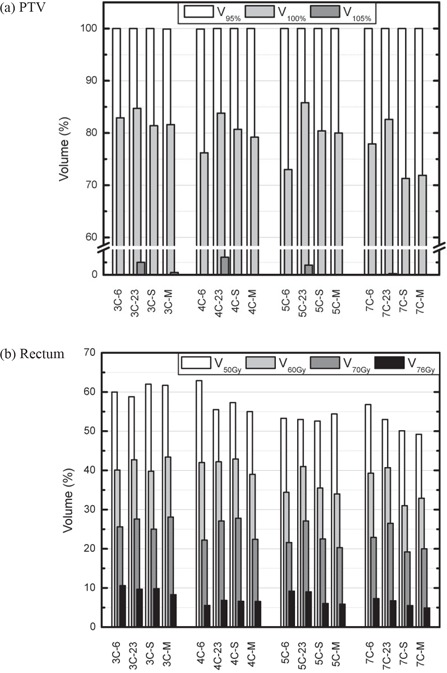
Planning target volume (PTV) coverage (a) and rectum sparing (b), for each optimized plan, in the prostate case P1. Plans are grouped by number of incidences. ($V_{x}$: volume receiving at least $x\;\text{Gy};\;V_{y\%}$: volume receiving at least *y*% of the prescription dose. See text for details on plan nomenclature.)

Figure 6 compares dosimetric indices for both the PTV and the rectum. Globally, $V_{95\%}$ values stayed the same for all plans. Values for $V_{100\%}$ varied between plans, being higher at 23 MV and lower when optimizing energy. Overdosages of the order of 105% were virtually absent. Regarding dose to the rectum, results varied greatly but tended towards a better sparing, with more incidences and energy optimization.

When averaged (±standard deviation(SD)) over all number of beam incidences, 95% CI values were 1.56±0.02(6MV),1.69±0.04(23MV),1.56±0.05(SEO), and 1.55±0.06 (MEO), while 100% CI values were 0.86±0.03 (6MV), 0.99±0.03 (23MV), 0.87±0.07 (SEO), and 0.86±0.06 (MEO). These plans also showed similarly heterogeneous dose distributions inside the PTV, which can be assessed by the HIs, having average ± SD values of 1.05±0.01 (6MV), 1.07±0.01 (23 MV),1.06±0.01 (SEO), and 1.06±0.01 (MEO).

Cases P2 and P3 consisted of five coplanar incidences, with the same segment generation scheme as case P1. The best results were for the 6 MV and the MEO plans. Improvements in CF value when adding energy optimization were of 24.4% (P2) and 5.1% (P3). Low energy was predominant for the MEO plans, with a proportion of 70.4% and 66.6%. Maximum dose to the ROB region decreased from 54.2 to 52.2 Gy (case P2) and from 55.6 to 53.9 Gy (case P3). Rectal dose remained under the dosimetric thresholds. Coverage increased in volume for case P2, with 95% CI values increasing from 1.52 to 1.55, and 100% CI values going from 0.977 to 1.02. For case P3, 95% CI values increased from 1.43 to 1.45, but the 100% CI values remained the same at 0.978.

### C. Lung case

For case L1, a plan was optimized for each of the four energy patterns and all four beam configurations (3C, 4C, 5C, and 7C). Results are presented in Table 4. The CF values for energy‐optimized plans were usually lower than the monoenergetic plans, except for the 5C configurations. When comparing the best of 6 or 23 MV with the best of SEO or MEO, the reduction of CF values was 10.1% (3C), 22.8% (4C), and 20.3% (7C). An increase of 15.0% was seen instead for the 5C plans. For half the cases, the MEO plan was the best polyenergetic plan in terms of CF value. In SEO, the proportion of MUs provided by the low‐energy beams generally increased with the number of incidences while in MEO, the ratio was more or less constant, with the exception of the 3C‐MEO plan.

**Table 4:** Cost function (CF) values, number of segments (Seg), and number of monitor units (MU) for the energy optimization in the lung case L1.

			*6 MV*		*23 MV*		*Total*	
*Confg.*	*Trial*	*CF*	*Seg*	*MU*	*Seg*	*MU*	*Seg*	*MU*
3C	6 MV	4.50003	4	305 (100.0%)			4	305
	23 MV	2.37508			8	274 (100.0%)	8	274
	SEO	2.18283	1	67 (24.1%)	6	211 (75.9%)	7	278
	MEO	2.13452	2	45 (16.2%)	7	233 (83.8%)	9	278
4C	6 MV	1.51573	7	310 (100.0%)			7	310
	23 MV	1.51833			9	274 (100.0%)	9	274
	SEO	1.17056	3	132 (46.0%)	6	155 (54.0%)	9	287
	MEO	1.40154	3	126 (44.5%)	5	157 (55.5%)	8	283
5C	6 MV	0.96749	8	310 (100.0%)			8	310
	23 MV	1.33405			10	267 (100.0%)	10	267
	SEO	1.22272	2	189 (64.1%)	6	106 (35.9%)	8	295
	MEO	1.11243	5	189 (66.1%)	2	97 (33.9%)	7	286
7C	6 MV	0.82299	8	308 (100.0%)			8	308
	23 MV	1.17814			9	262 (100.0%)	9	262
	SEO	0.65619	8	180 (62.7%)	3	107 (37.3%)	11	287
	MEO	0.82113	4	143 (51.5%)	5	137 (48.9%)	9	280

**Figure 7:**
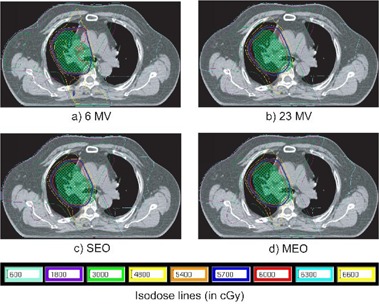
Isodose distributions for a 3‐coplanar beam configuration and for each type of energy optimization, in the lung case L1. The green structure is the PTV.

**Figure 8:**
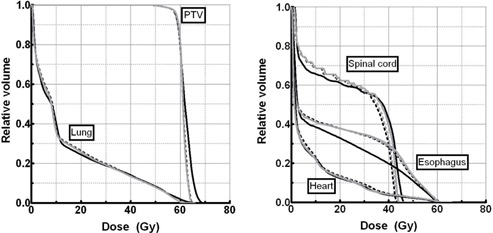
Dose‐volume histograms for a 3‐coplanar beam configuration and for each type of energy optimization, in the lung case L1. (solid black: 6 MV; dashed black: 23 MV; solid grey: SEO; dashed grey: MEO)

Isodose inspection of 3C plans (see Fig. 7) shows that at 6 M V, the 48 Gy (80%) isodose covered more area and that there was an overdosage of over 57 Gy (95%) in the posterior region. This was not seen for more incidences. The DVHs for these four plans are presented in Fig. 8. The 3C‐6MV plan delivers higher dose to the PTV but is comparable in terms of $V_{100\%}$ with the other plans.

**Figure 9:**
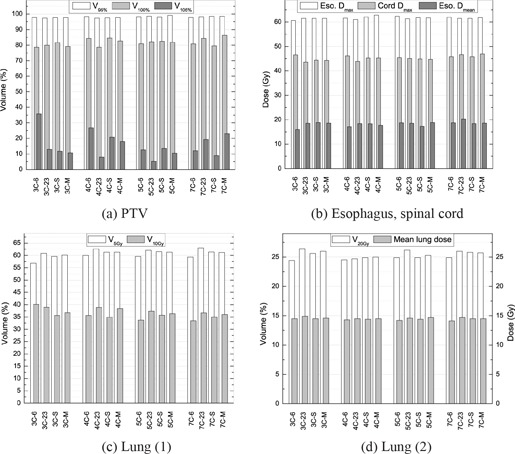
Planning target volume (PTV) coverage (a) and organs at risk sparing (b)–(d), for each optimized plan, in the lung case L1. Plans are grouped by number of incidences. ($V_{x}$: volume receiving at least $x\;\text{Gy};\;V_{y\%}$: volume receiving at least *y*% of the prescription dose; MLD: mean lung dose; $D_{\max}$: maximum dose; $D_{\text{mean}}$: mean dose. See text for details on plan nomenclature.)

Dosimetric indices can be compared using Fig. 9. While $V_{95\%}$ values did not improve much on average, $V_{100\%}$ went up about one point (81.2% 6 MV, 81.3% 23 MV, 82.1% SEO, 82.6% MEO). Values of $V_{105\%}$ with energy optimization were between those for monoenergetic plans, showing an improvement relative to the 6 MV plans (21.8% 6 MV, 11.3% 23 MV, 13.8% SEO, 15.6% MEO). For esophagus and spinal cord, variations on average $D_{\max}$ and $D_{\text{mean}}$ (for the esophagus) were less than one point. Lung sparing was similar between plans, according to the $V_{20\text{Gy}}$ and the mean lung dose (MLD), but $V_{10\text{Gy}}$ values decreased slightly when adding incidences. However, $V_{5\text{Gy}}$ did the opposite, and increased sensibly with an increasing number of incidences.

Plans produced for each optimization pattern could be considered similarly conformal. The 95% CIs were, when averaged (±SD) over all number of incidences, (±SD) (6 MV), 1.27±0.04(6MV), 1.24±0.02(23MV),1.25±0.01(SEO), and 1.24±0.01 (MEO). For their part, 100% CIs were 0.85±0.05(6MV),0.81±0.02(23MV),0.82±0.04(SEO), and 0.81±0.03(MEO). Heterogeneity was globally equivalent between plans: the average ±SD values were 1.11±0.02 for 6 MV and 1.09±0.01 for the other three energy patterns.

Cases L2 and L3 used four beam incidences. In both cases, the best monoenergetic plan was obtained at 23 MV, and the best polyenergetic plan involved MEO. Improvements in CF values were of 23.8% (L2) and 22.6% (L3). Proportions between energies varied, with 48% of 6 MV for case L2 and only 18.3% for case L3. An increase in $V_{95\%}$ was seen for both cases, by an amount of 0.5% and 2.2%, respectively. HI values remained at 1.12 for case L2 but were improved for cases L3 (from 1.16 to 1.11). Computed OAR indices did not vary much, always remaining under tolerance baselines.

## IV. DISCUSSION

In this study, we have described the implementation of two methods for beam energy optimization in the inverse planning system Ballista, impacting on either a stochastic or a deterministic method. Although it was applied to 6 and 23 MV beams only in this study, it can be easily generalized to a higher number of energies available. Also, even though some of the results obtained could have been expected to some extent, we are not aware of other studies that formally showed this in a multi‐energy inverse planning optimization context.

The rationale behind using two optimization engines goes back to the initial conception of the system.^(^ ^7^ ^,^ ^8^ ^)^ Orientation and weights optimization problems can be dealt with in a completely separate manner or in conjunction with one another. Changing the number of variables treated in one algorithm does not affect the speed of the second algorithm. Therefore, SEO and MEO methods for energy optimization do not impact on the whole system but only on the part where they apply. Since stochastic algorithms are already time‐consuming and adding variables commands a more exhaustive search of solution space, the SEO method can be considered more unstable, especially when cooling is hastened. On the other hand, the MEO method relies on a deterministic algorithm which, by definition, has to produce the same solution every time it is run. Increasing the number of variables can, however, slow down the convergence towards the optimal solution. When optimizing beam orientations at the same time as energy in MEO mode, this effect is repeated at each iteration of the FSA process. We have seen, in our study, that the MEO method has a greater impact on global optimization speed. The BCQN engine, as currently implemented within the OPT++ library, is less effective when increasing the number of variables and using a quadratic cost function. To circumvent this problem, efforts are currently being made toward linear programming, using strategies such as those introduced by Romeijn et al.^(42)^

Results show that for our prostate case, the use of low‐energy beams becomes more relevant when adding beam incidences, since the effect of high entry dose is diluted. This leads to the interesting conclusion that one can treat tumors located deep inside the body while using some – or even exclusively – low‐energy beams. The number of MUs for 6‐MV‐only plans is always greater, but otherwise lacks the undesirable neutron dose given by high‐energy beams. Our results for aperture‐based IMRT agree with the work by Sun and Ma^(18)^ who showed that low energy is a feasible choice of the treatment of prostate cancer with simple IMRT with limited modulation.

Lung cancer cases can usually be treated with as low as three beam incidences, without compromising target conformity. Using too many incidences produces wider low‐dose regions, which can be harmful to the lungs, especially in the context of concurrent chemotherapy.^(43)^ Radiation pneumonitis has been frequently correlated with many forms of dosimetric evaluations (normal tissue complication probability (NTCP), $V_{\text{dose}}$ and MLD) and complications are often linked with low dose deposited in the whole lung volume. Many of these studies are reported in a systematic review by Rodrigues et al.^(44)^ Energy optimization, in the present case, brings incremental gain to the overall quality of the treatment plans, particularly with many beams. In some cases, the gain is translated in lower MUs for a similar or better CF value. Our results are in agreement with previous findings^(^ ^21^ ^–^ ^23^ ^)^ stating that low‐ and high‐energy beams lead to similar dose distributions. We found that the advantage of optimizing energy lies primarily in the decrease of MUs with high energy while maintaining the conformance given by low energy. Differences in dosimetric parameters used to assess complication probabilities are not necessarily significant clinically. However, we believe that even the slight improvement brought by energy optimization, combined with the decrease in monitor units and preservation of conformity, contribute to the design of a preferable treatment plan.

The problem of beam energy is clear for thoracic irradiations, because the lungs are a low‐density medium where electronic disequilibrium is likely to occur. Beam penumbra tends to be wider than in average tissue, which complicates target conformity and OAR sparing. Dose calculation engines have to take into account this phenomenon in order to be accurate. Should the dose calculation model be erroneous, any result produced by the optimization will be wrong as well. Pencil‐beam algorithms are, therefore, not suited for this task, while collapse‐cone and, preferably, Monte Carlo methods can achieve the necessary accuracy.^(45)^ As was mentioned before, in this study we have used the well‐established superposition/convolution method, which can accurately account for density variations.^(30)^

From a clinical point of view, an approach similar to MEO can be easily implemented in forward planning: the planner can manually try different energies to fulfill the prescription and tolerance doses. However, this method is not optimal in a numerical sense, as an inverse planning system is most likely to find a better and potentially non‐intuitive solution. The AB‐IMRT approach ensures a limited increase in plan complexity compared to standard, forward‐planned 3D‐CRT. An important advantage of using the Ballista system is that energy can be optimized at the same time as beam angles, a feature that is not present in most commercial planning systems. To achieve a similar result, a planner has to specify an unnecessarily high number of incidences; even so, non‐coplanar configurations are usually not explored. Furthermore, combining beam energies could be seen as similar to using a beam with an intermediate energy, an option that is not always available to every clinic. This will be explored for potential dosimetric benefit for lung tumors in future investigations.

## V. CONCLUSIONS

For deep‐ and moderately deep‐seated targets, treated using AB‐IMRT, there is an interest in optimizing the energy to benefit from both low‐ and high‐energy dose deposition characteristics. The use of fewer fields increases the potential of energy optimization in order to avoid dose in regions distant from the target volume (close to the skin surface). It has been shown that deep‐seated targets can be treated at low energy without degrading plan quality. In low‐density media, such as lung tissue, mixing energies can compensate for lateral diffusion of the dose distributions and allow balance over number of monitor units and target conformity. Beam energy optimization could find its use in many contexts; for example, in a dose escalation protocol, where higher dose would need to be deposited in depth and target conformity would be more critical because of surrounding sensitive structures.

## ACKNOWLEDGMENTS

This work is supported by the Natural Sciences and Engineering Research Council of Canada (NSERC). We acknowledge Philips Medical Systems for providing a research version of the Pinnacle^3^ treatment planning system. Contributions from Dr Julie Harvey for the literature review on rectum dose‐volume parameters and from Dr François Germain for manuscript review are also acknowledged. J.S. would like to thank the NSERC for financial support through a postgraduate scholarship.
